# Mitochondrial lineages in *Notochthamalus scabrosus* as indicators of coastal recruitment and interactions

**DOI:** 10.1002/ece3.283

**Published:** 2012-07

**Authors:** Kelly M Laughlin, Christine Ewers, John P Wares

**Affiliations:** Department of Genetics, University of GeorgiaAthens, Georgia 30602

**Keywords:** Cline, *Notochthamalus*, phylogeography, source-sink, temporal

## Abstract

A significant genetic cline has previously been identified along the Chilean coast in the barnacle *Notochthamalus scabrosus*. Samples from the previous study, spanning 800 km, were not able to show whether the southern lineage ultimately goes to fixation at higher latitudes. In addition to expanding the geographic sampling of this species, locations that were sampled approximately four to five generations ago were resampled for this study, enabling a temporal comparison of the location and strength of the observed cline. Here, we show that although the cline persists as expected, the tremendous changes in observed lineage frequencies near the clinal boundary are indicative of source-sink dynamics that may be associated with a codistributed biogeographic transition zone. We also find that the southern lineage does not increase in frequency in more southern populations, suggesting that this lineage is maintained through a combination of density-dependent interactions and a coastal fitness gradient.

## Introduction

Ocean currents have been shown to be key drivers of benthic marine diversity (Wares et al. 2001; Waters and Roy 2004; White et al. 2010). This relationship is largely because of the tendency for many marine organisms to reproduce via larvae that must feed suspended in the water column for many days or weeks. Thus, currents can act as mechanisms of transport (Gaylord and Gaines 2000; Becker et al. 2007) or disruption (Rocha-Olivares and Vetter 1999; Barber et al. 2002; Waters and Roy 2004) among locations. However, patterns of migration do not necessarily predict patterns of gene flow. Sotka et al. (2004) show that although reduced transport by coastal currents may be involved, it is likely that the strong multilocus cline found in the intertidal barnacle *Balanus glandula* is maintained by other factors, including natural selection (Pringle and Wares 2007).

*Balanus glandula* has been intensely studied in part because the behavior of genetic diversity in that species has been thought to be a good indicator of how other species with similar larval life histories would respond to the oceanic environment (Wares and Castañeda 2005). The Pacific coast of North America has a number of significant and well-studied biogeographic (Burton 1998; Dawson 2001; Wares et al. 2001; Pelc et al. 2009) and ecological (Connolly et al. 2001) transitions that are at least in part mediated by interactions of coastal dynamics such as upwelling and the strong equatorward California Current. However, the concordance of how all of these factors influence coastal diversity is not yet well understood.

What is unusual still in the field of biogeography, and necessary to put such factors together in a meaningful way, is any sort of system-wide replication (Haydon et al. 1994). Through evaluating very similar systems, we may gain greater insight into what coastal and oceanic features are most important for promoting and maintaining marine diversity (Fernández et al. 2009). The comparison between the Pacific temperate coasts of North and South America is an intriguing one. Each coast harbors a phylogenetically and ecologically similar intertidal biota, is strongly influenced by an equatorward current (the Humboldt Current System in the south; Thiel et al. 2007), has clear latitudinal shifts in the strength and persistence of coastal upwelling (near Cape Blanco, 42°N, along the North American coast; near Coquimbo, 30°S, along the South American coast), and transitions in density-dependent community effects associated with these latitudinal patterns (Navarrete et al. 2008). Both coasts are topographically relatively simple, with intense research programs relating to coastal currents, shelf topography, fisheries management, and benthic/intertidal diversity.

Zakas et al. (2009) showed an intriguing concordance between one of the primary transitions along the Chilean coast, near 30°S—where upwelling, ecology, and a number of indicators of marine productivity transition along with a major biogeographic transition (Camus 2001; Navarrete et al. 2005; Thiel et al. 2007)—and a cline in mitochondrial diversity in the intertidal barnacle *Notochthamalus scabrosus*. Essentially, across an approximately 800 km survey of the Chilean coast, two primary lineages at the mitochondrial cytochrome oxidase I (mtCOI) locus were distinguished, with one group (the “A clades”) found throughout the domain surveyed, and the other (the “B clade”) being a divergent (by about 2%) haplotype group primarily only found in populations from Punta Talca (31°S) southward. From this study, several questions remained. First, given the latitudinal concordance with bio-geographic transitions along the coast, does the phylogeography of *N. scabrosus* represent a true transitional cline that reflects the same mechanisms involved in the biogeographic transition? Or, are more complex mechanisms involved that may confound the association of the detected cline with nearshore biogeography (Burton 1998)?

The latitudinal transition in haplogroup diversity in *N. scabrosus* is comparable to that seen in *B. glandula*, except that without sampling a greater geographic range it is difficult to know whether, for example, the southern “B” haplogroup goes to fixation in samples from locations further south, as in *B. glandula* where the two primary haplogroups approach fixation at the latitudinal extremes. It is also not clear how spatially or temporally stable this pattern is. Here, we extend the mitochondrial survey of *N. scabrosus* to approximately 2300 km of the Chilean coastline, allowing confirmation of the initial survey as well as evaluation of the temporal stability of haplogroup frequencies at sites close to the central transition zone. We include samples from different intertidal microhabitats as well, in consideration of potential niche differentiation (Boaventura et al. 2002; Wares and Castañeda 2005).

Given that the primary haplogroups were previously identified and characterized (Zakas et al. 2009), we also evaluate the widely distributed A haplogroups as a subset of the total sample of genetic diversity. This approach is taken so that we can better understand the degree to which these lineages interact: is *N. scabrosus* a single evolutionary species with disruptions in gene flow in the middle of its range caused by changes in transport and selection gradients? Or two possibly interacting but somewhat independent evolutionary groups? It is not yet clear how easy it is to make predictions about species interactions and genetic diversity in a community, though in many cases an association can be shown (Hughes and Stachowicz 2004; Vellend 2005; Johnson and Stinchcombe 2007; Hughes et al. 2008). Primarily, we evaluate the A lineage on its own to avoid the confounding analytical effects of including a highly divergent set of haplotypes that are not equally distributed.

Overall, our goals are to better understand the distribution of mitochondrial diversity in *N. scabrosus* and how current-driven gene flow patterns are involved in the maintenance of this diversity. Having samples from multiple time periods enables us to ask specifically whether the shift in relative abundance of the lineages observed at Punta Talca is stable; our working hypothesis is that this site and locations to the north may represent unsuitable habitat for consistently supporting the southern lineage. Also, our samples from locations further south than in Zakas et al. (2009) enable us to test the hypothesis that the southern lineage goes to fixation at higher latitudes.

## Methods

**Figure 1:**
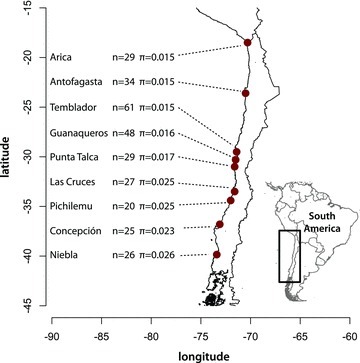
Sample locations are indicated along the coast of Chile, with mitochondrial haplotype sample size and overall nucleotide diversity (π) indicated.

Specimens of *N. scabrosus* were collected in 2010 and 2011 from intertidal habitats at the locations indicated in Figure 1. At two locations (Temblador and Guanaqueros), specimens were collected from the “high” and “low” regions of the intertidal zone in which *Notochthamalus* is found (typically in the portion of the intertidal zone between the upper distribution of marine organisms and the upper bound of the *Perumytilus* zone). Individuals were collected directly into labeled tubes of 95% ethanol.

DNA was isolated using the same dissection and isolation protocols as in Zakas et al. (2009). Similarly, PCR and sequencing of the mtCOI gene region followed the same protocols. Sequences were edited and aligned using Codoncode Aligner version 3.7.11; each sequence was first checked for taxonomic match with previous *N. scabrosus* data using BLASTn, and then edited for sequence quality. Nucleotide calls with quality scores <20 (after assembly of forward and reverse reads) were assumed ambiguous and coded as such. Sequences with fewer than 400 unambiguous nucleotides (from an amplified fragment length of 710 bp) were removed from the dataset. Phylogenetic analysis of these data was performed using BEAST v1.6.1and TreeAnnotator v1.6.1 (allowing for rate variation under a HKY model of evolution, with MCMC chain length of 10^7^ sampled every 1000 trees and enforcing a strict clock), using voucher sequences from Zakas et al. (2009) to identify the A1, A2, and B clades and see if additional haplogroups warranted analysis in terms of spatial frequency.

After each sequence and population was characterized with respect to haplogroup composition, overall and within-haplogroup molecular population genetic parameters (π, Tajima's D, pairwise *F*st) were estimated using Arlequin version 3.5.1.2 (Excoffier et al. 2005). Pairwise *F*st values were used to test for isolation by distance within the A clade sequences only, using standard approaches and 1000 Mantel test permutations. As in Zakas et al. (2009), we employed a serial grouping of regional populations into two groups for analysis of molecular variance (AMOVA) to explicitly address whether the A clade alone reflects any phylogeographic break that could be associated with either known bio-geography or the presence/absence of the B clade. Our primary focus was on finding the grouping that maximized *F*ct as in Dupanloup et al. (2002) but other AMOVA-based *F* statistics were evaluated for potential information about genetic structure.

We further investigated whether the pattern of haplotype diversity within the A group can be best explained by latitude or by potential interactions with the B clade. Correlation analysis between haplotype diversity h(A), latitude, and frequency of B was performed to identify correlated parameters. We then conducted linear regression analyses between latitude and h(A) or frequency of B and h(A). We chose frequency of B rather than haplotype diversity of B because of the large error of haplotype diversity associated with small sample sizes. AICc (Akaike information criterion with a correction for finite sample sizes) values and weights were calculated for three models: latitude explains h(A), freq(B) explains h(A), or no parameter explains h(A) (intercept-only model). Studentized residuals and Cook's D were used to identify outliers. These were excluded in a repeat analysis.

Our goal with assessing population size and migration rates among sampled locations is to determine whether mean current direction—specifically, the northward flow of the Humboldt Current—is a significant factor in shaping diversity within the A haplogroups of *N. scabrosus* (Zakas et al. 2009 showed that this relationship changes when the B group is included). Data were analyzed with Migrate version 3.2.16 (Beerli 1997), with a specific goal of using comparison of multiple runs via log Bayes factors (LBF) to identify the most appropriate model (Beerli and Palczewski 2010). Following a series of preliminary runs to determine appropriate ranges of theta and migration to include in the prior distribution, we found that theta at each location was typically <0.1 and used an exponential prior ranging from 0 to 0.2. As the goal of this analysis is to compare the potential for asymmetric gene flow, we directly compare the posteriors of analysis in which pairwise migration parameters are directionally independent (i.e., from population 1 to population 2 is not the same as from 2 to 1) to analyses in which the migration is forced to be symmetric between each pair of locations, using the LBF test. This takes the log likelihood of each result, with all other components of the MCMC search identical, and the difference of these likelihoods indicates the strength of support for one hypothesis over the other.

As preliminary analyses also suggested some sensitivity of this test to the shape and size of the prior distribution for M, two sets of exponential priors were chosen for M. In “small migration” models, the prior of M is exponentially distributed from [0,100]. The “large migration” models have a prior from [0,1000]. Although in both cases there is potential for evolutionarily enormous migration rates, these two scenarios were chosen from an infinite number of scenarios to query the sensitivity of the LBF test on the prior. If asymmetry is significantly better than symmetry in both cases, we can proceed in describing the concordance of asymmetric flow with nearshore current flow, and discuss the effect of the prior on the magnitude of estimated M. If symmetry is better than asymmetry, we can proceed with less concern about the association of gene flow with large-scale currents and focus on the magnitude of M. If the two sets of runs disagree, we assume (again, following the results of preliminary analyses) that our data may not be informative enough to overcome the assumptions of the prior on this parameter.

Of these four scenarios (two prior ranges, symmetry vs. asymmetry), we permitted a fully parameterized analysis of theta and migration. Initial attempts included strict stepping-stone models of migration (parameter only estimated between neighboring locations); however, as our data do not fit a pattern of isolation by distance (see Results) and the geographic scale of location pairs varies across the dataset, there are an enormous number of potential models to be explored. As our focus is simply on whether or not to proceed considering asymmetric flow, we ask only whether asymmetry is a model favored over symmetry among pairs of locations. Also, as the data do not fit a pattern of isolation by distance under traditional analyses, we do not use a geofile to scale migration rates by geographic distance.

We interpret the output of our two pairs of analyses using LBF within each pair, and then whether this hypothesis test produces an equivalent result in both sets of assays (asymmetry vs. symmetry). Only if both migration priors are consistent do we proceed with interpretation of the quantitative results. All analyses used a DNA sequence model assuming a 2:1 transition:transversion ratio with site rate variation, a random start genealogy and estimates of theta and M based on *F*st, and subsampling of datasets to select 10 random individuals from each location as advocated in Wares and Cunningham (2005). “Slice” proposal distribution was employed, with exponential priors on theta and migration as above. For Bayesian inference, a single long chain is recommended (Migrate manual) and was used in all cases with sample increment of 20 and 5,000,000 recorded steps in the chain, with a burn-in of 100,000 steps. Four independent heated chains were used as replicates, with an exponential heating (from 1 to 10) scheme. A pairwise convergence diagnostic was calculated. Each of these four replicated analyses was independently run twice on a Mac Pro with a 2.66GHz quad-core processor and 24 GB of RAM.

## Results

**Figure 2:**
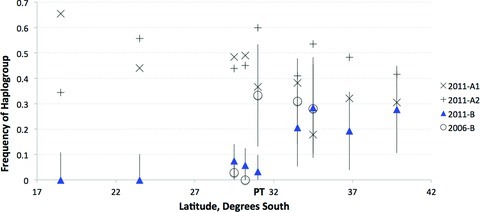
Observed frequencies of haplogroups, as identified through Bayesian clade probabilities, across the geographic range of *N. scabrosus* as sampled. Haplogroup B data are shown from 2006 (Zakas et al. 2009) for the locations shared with current (2010–2011) sample. Error bars are based on binomial sampling frequencies and are shown only for the B clade samples for clarity. Only Punta Talca (labeled “PT” on horizontal axis) exhibits a significant (*P* < 0.05) shift in frequency of B clade individuals. All other changes in haplogroup frequencies are not statistically significant.

Sequence data (Genbank JQ950750–JQ951089) are summarized in Figure 1 to show the sample size and nucleotide diversity across all sequences (both haplogroups) at each location. The A1, A2, and B clades identified in Zakas et al. (2009) were recovered in the current sample, with each clade having a posterior probability of 0.97 or higher (not shown). No additional subclades of more than 10 individuals were supported with similarly high posterior probability. The frequency and binomial-estimated sampling error of haplogroups A1, A2, and B at each location were estimated and are shown in Figure 2. Only Punta Talca exhibits a significant (*P* < 0.05) shift in the frequency of B between samples from 2004 to 2006 (Zakas et al. 2009) and our current samples. As we know from our previous study (Zakas et al. 2009) that the signals of within- and among-population structure and migration are influenced by the presence of B in the dataset, all following analyses focus only on the dynamics of the A haplogroup.

Excluding the B clade, values of Tajima's D range from –1.480 (Pichilemu) to 0.003 (Valdivia), with no samples deviating significantly (average –0.793, standard deviation [SD] 0.518) from null expectations. Isolation by distance across our samples was not supported (*r* = 0.278, *P* = 0.187). Overall structure within the A lineages was minimal across the range of *N. scabrosus*, with no *F*ct value greater than 0.02 (ns) with the exception of grouping the two northernmost populations (Arica, Antofagasta) as separate from the rest of the domain (*F*ct 0.059, *P* = 0.026). Given the geographic distance separating these sites from the rest and the effect of multiple contrasts of our data, the biological conclusions to be made from this pattern are slight. No significant *F*st values were identified between high and low intertidal samples at Temblador and Guanaqueros.

**Figure 3:**
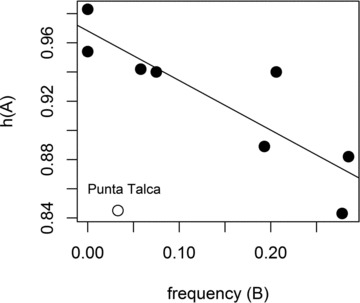
Linear regression of haplotype diversity in the A lineages at each sampled location against the 2010–2011 frequency of the B haplo-group at that location. The data for Punta Talca from 2010–2011 are shown in white and are statistically identified as an outlier (note the observed frequency for Punta Talca in 2004–2006 was 0.33); exclusion of this data point generates a significant relationship in which the frequency of B has a higher Akaike weight (0.70) than latitude in explaining h(A), though the frequency of B and latitude are also correlated.

From our linear regression analysis, the frequency of B and latitude are correlated (–0.85, *P* < 0.01) and so were not used in linear regression analysis together. As the correlation is not perfect, we analyzed them separately using an AICc framework to identify which variable has a higher relative probability of explaining h(A). The data point from Punta Talca was identified as an outlier (Studentized residual = –4.1935, Cook's D = 0.6156). In the full analysis (Punta Talca included), latitude was the best variable to explain h(A) (AICc weight 0.63). Excluding Punta Talca made the frequency of B a better variable for explaining haplotype diversity of A (AICc weight 0.7; Fig. 3).

Analysis of gene flow using Migrate required approximately 10 days computational time for each of the eight runs indicated. Convergence of chains was assured through assessment of the Gelman indices as approaching 1, and the effective sample size for parameter estimates was generally greater than 500. For three of our four pairs of runs (excluding one of the runs of asymmetry vs. symmetry “small”), calculation of the log marginal likelihood (lmL) obtained from thermodynamic integration of multiple chains for each proposed model strongly supported (LBF ≤–9.8, relative to the symmetric lmL) the symmetric migration case. In one of the paired runs (“small” migration limits), asymmetric migration was strongly supported (LBF 27.69) over symmetric migration. An additional strategy employing 50 MCMC chains of shorter length led to similarly ambiguous results. The tendency toward supporting the more limited parametric case (symmetry being a special case of asymmetry), but in-consistency of all runs, leads us to conclude that there is not strong support for asymmetric gene flow.

For most migration parameters estimated in these analyses, the mode estimate was close to the prior boundary (whether “small” or “large,” as in Methods), indicating our single locus provided insufficient information to sufficiently resolve within the analyzed parameter domain. Previous analyses (Zakas et al. 2009) had suggested that the migration rates were extremely large among populations for these data, and that convergence on differential rates would be difficult to obtain. Effectively, our results (not shown) indicate that migration is extremely high among all pairs of sites, making the biological meaning of symmetry versus asymmetry very minor in any case. Estimates of θ (2*N*eμ) were relatively stable across the nine sites, with mean 0.099 ± 0.034 allowing migration to be large, and 0.059 ± 0.04 in the small migration scenario. No single location was consistently more than one SD from the mean in either case.

## Discussion

While many marine clines have been documented, the temporal and spatial stability of those clinal patterns is shown in fewer studies (Hilbish 1985; Hare and Avise 1996; Schmidt et al. 2008). The differentiation of northern and southern populations of *B. glandula*, for example, is temporally stable and centered in north central California (Wares et al. 2001; Sotka et al. 2004; Galindo et al. 2010), though with significant interannual variation in haplogroup frequencies at some locations (Barshis et al. 2011). Here, we show that the cline indicated in Zakas et al. (2009) is stable following three to four generations of reproduction and a major El Niño event, but note two key observations: first, the cline is incomplete in that the southern “B clade” type never rises above a frequency of 30% in our samples, even as our samples continue another approximately 500 km to the south of previous samples; second, that the temporal shift in haplogroup frequency near the edge of the cline (specifically, at Punta Talca) is significant and suggestive of the metapopulation dynamics governing this pattern.

Punta Talca exhibited an order of magnitude reduction in frequency of B clade haplotypes between samples taken in 2004–2006 and samples collected in 2010 (Fig. 2). Overall, the frequency shifts in other haplogroups were large at this location as well (a change of 27% in the A2 group, for example). Although data from a single locus are insufficient for using temporal methods to estimate effective population size (Waples 1989), these shifts are consistent with an extremely low effective population size at this location, significant immigration, or both. Punta Talca is recognizably lower in barnacle density and recruitment than many surrounding sites (Broitman et al. 2001) and experiences strong, persistent upwelling. These factors suggest that the downstream (northward) edge of the B clade distribution could be a demographic sink (Pulliam 1988; Wares and Pringle 2008). However, neither π, haplotype diversity, nor the number of singleton haplotypes are significantly reduced at this site, as might be expected in a demographic sink (though the expectations of diversity at “downstream” or “sink” locations are notoriously difficult to derive without much additional information; Dias 1996; Wang and Caballero 1999; Wang and Whitlock 2003). With extensive upwelling, self-recruitment (retention, sensu [Pringle and Wares 2007]) may be a key factor in maintenance of allele frequencies at Punta Talca (Thiel et al. 2007) and nearby biogeographic transitions.

Few additional insights are gathered from analysis of the A haplogroups alone. There is little phylogeographic structure to these data, and high and homogeneous migration inferred across this broad geographic range. Given that there are still questions about the behavior of *Notochthamalus* larvae in the pelagic larval phase, the degree to which larvae would be expected to entrain in the northward Humboldt Current or the southward Peru Undercurrent is not clear (Aiken et al. 2011); there is of course considerable stochasticity in nearshore current flow and larval transport. From the dispersal of A, it seems we learn little about the geographic limits on the distribution of B. So far we only know that the B lineage has not yet been recovered north of the Temblador site, and—peculiarly—there is a statistical interaction between the diversity of haplotypes within the A lineages and the proportion of individuals of *N. scabrosus* at a given location that carry the B lineage (Fig. 3).

Though work to date suggests that *N. scabrosus* is effectively not in competition for resources with the confamilial and similarly distributed *Jehlius cirratus* (Shinen and Navarrete 2010), there are few predictions about what to expect for genetic diversity patterns between lineages that are in competition (Johnson and Stinchcombe 2007; Hughes et al. 2008; Robinson et al. 2009; Örjan 2011). In one of the only direct tests of whether density-dependent effects among genotypes or clones leads to patterns of diversity, Örjan (2011) found that other stochastic demographic factors are likely to have stronger effects; however, the geographic scale and evolutionary age of the patterns identified here in *N. scabrosus* are considerably greater and may involve larger shifts in effective population size of each haplogroup. Understanding whether the interaction illustrated in Figure 3 is meaningful may be important for understanding how competition structures this community, and how these dynamics will change along with the climate (Aiken et al. 2011).

However, the boundary of the B clade is also likely to be defined by some form of natural selection, such as postsettlement mortality (Shinen and Navarrete 2010). For a typical cline, we might expect the B clade to go toward fixation in samples taken further south (but see Wares and Cunningham 2005); instead, it never is found at frequency greater than 30%. While additional information may be necessary from populations of *N. scabrosus* still further south (it is found as far south as Beagle Channel in southern Argentina; (Curelovich et al. 2009), the behavior of this evolutionary lineage is much like a cryptic species with more restrictive physiological tolerances (Schizas et al. 2001). Sequence data from a single nuclear marker (elongation factor 1-alpha) suggested that the major haplogroups in *N. scabrosus* are randomly mating (Zakas et al. 2009); additional data from the nuclear genome will be necessary to evaluate the potential mechanisms maintaining the distributional edge of this lineage.

What this study tells us is that an unusual combination of environmental and evolutionary factors are involved in maintenance of the extraordinary mitochondrial diversity in *N. scabrosus*. Additional spatial and genomic surveys will be necessary to tease apart the characteristics of the distribution of this diversity, and experimental work is planned to identify the extent to which postsettlement mortality, differential transport, and/or competition could be involved in the observed patterns. What we have shown here is an unusual temporal assessment of the stability of a large-scale genetic cline, giving insights into potential metapopulation dynamics of the system. The system itself is potentially useful as a biogeographic replicate of the Pacific coast of North America, and the extensive research into community ecology and population genetics on both coasts enables further insight as we improve our understanding of the physical drivers of gene flow. Finally, though speculative, this study illuminates the potential for density-dependent interactions to influence levels of genetic diversity, an effect that could expand the current range of molecular ecology studies.
